# Assessing the Stroke-Specific Quality of Life for Outcome Measurement in Stroke Rehabilitation: Minimal Detectable Change and Clinically Important Difference

**DOI:** 10.1186/1477-7525-9-5

**Published:** 2011-01-19

**Authors:** Keh-chung Lin, Tiffany Fu, Ching-yi Wu, Ching-ju Hsieh

**Affiliations:** 1 School of Occupational Therapy, College of Medicine, National Taiwan University, 17, F4, Xu Zhou Road, Taipei, Taiwan; 2 Division of Occupational Therapy, Department of Physical Medicine and Rehabilitation, National Taiwan University Hospital, 7 Chung-shan South Road, Taipei, Taiwan; 3 Department of Occupational Therapy and Graduate Institute of Clinical Behavioral Science, Chang Gung University, 259 Wenhua 1st Road, Taoyuan, Taiwan; 4 Institute of Biophotonics, National Yang-Ming University and Department of Ophthalmology, Taipei City Hospital-Heping Branch, Taipei, Taiwan

## Abstract

**Background:**

This study was conducted to establish the minimal detectable change (MDC) and clinically important differences (CIDs) of the physical category of the Stroke-Specific Quality of Life Scale in patients with stroke.

**Methods:**

MDC and CIDs scores were calculated from the data of 74 participants enrolled in randomized controlled trials investigating the effects of two rehabilitation programs in patients with stroke. These participants received treatments for 3 weeks and underwent clinical assessment before and after treatment. To obtain test-retest reliability for calculating MDC, another 25 patients with chronic stroke were recruited. The MDC was calculated from the standard error of measurement (SEM) to indicate a real change with 95% confidence for individual patients (MDC_95_). Distribution-based and anchor-based methods were adopted to triangulate the ranges of minimal CIDs. The percentage of scale width was calculated by dividing the MDC and CIDs by the total score range of each physical category. The percentage of patients exceeding MDC_95 _and minimal CIDs was also reported.

**Results:**

The MDC_95 _of the mobility, self-care, and upper extremity (UE) function subscales were 5.9, 4.0, and 5.3 respectively. The minimal CID ranges for these 3 subscales were 1.5 to 2.4, 1.2 to 1.9, and 1.2 to 1.8. The percentage of patients exceeding MDC_95 _and minimal CIDs of the mobility, self-care, and UE function subscales were 9.5% to 28.4%, 6.8% to 28.4%, and 12.2% to 33.8%, respectively.

**Conclusions:**

The change score of an individual patient has to reach 5.9, 4.0, and 5.3 on the 3 subscales to indicate a true change. The mean change scores of a group of patients with stroke on these subscales should reach the lower bound of CID ranges of 1.5 (6.3% scale width), 1.2 (6.0% scale width), and 1.2 (6.0% scale width) to be regarded as clinically important change. This information may facilitate interpretations of patient-reported outcomes after stroke rehabilitation. Future research is warranted to validate these findings.

## Background

Although the stroke mortality rate has been declining [1], the estimated prevalence rate of stroke-related disability is about 331 per 100,000 [2]. Stroke disability and morbidity cause reduced quality of life (QOL) among stroke survivors [3]. The greater the disability, the lower the QOL is [4]. With ongoing rehabilitation, however, improvements in functional status are possible [5] and contribute to increase QOL for stroke survivors. Therefore, the assessment of stroke rehabilitation should include disability and QOL domains, which are influenced by the disease [6-9].

Generic QOL instruments such as the Medical Outcomes Study Short-Form 36-item survey (SF-36) may underestimate the effect of stroke [10]; therefore, disease-specific tools are considered more helpful in providing information about the difficulties that patients with stroke may experience [7,11]. Because the information from the patients' perspective on the consequences of disease and the therapeutic benefits is considered critical in the evaluation of health care, patient-reported outcome measures have been used to supplement clinical decisions made from physician-based outcome measures [12]. Of the stroke-specific scales, the Stroke-Specific Quality of Life Scale (SS-QOL) [13], in addition to the Stroke Impact Scale version 3.0 (SIS 3.0) [14], is the most comprehensive [15] and frequently used patient-reported outcome measure [16-19].

The SS-QOL is a self-report questionnaire consisting of 49 items in the 12 domains of energy, family roles, language, mobility, mood, personality, self-care, social roles, thinking, upper extremity (UE) function, vision, and work/productivity. The domains are scored separately, and a total score is also provided. The psychometric properties of the SS-QOL have been validated in patients with ischemic stroke and intracerebral hemorrhage [10,18,20]. In patients with subarachnoid hemorrhage, the 12 SS-QOL domains and the total score demonstrated good internal consistency [21]. The SS-QOL items also have acceptable agreement with the categories of the International Classification of Functioning, Disability, and Health, which indicates that the SS-QOL covers multidimensional components meaningful for patients with stroke [22]. The clinical utility of the SS-QOL remains understudied, however, and several clinimetric properties, such as the minimal detectable change (MDC) and the clinically important difference (CID) of the SS-QOL, have not yet been investigated. This information helps inform clinical decision making on the discontinuation or alteration of a treatment program that aims to improve patients' physical function.

The MDC is the smallest change that can be detected by the instrument beyond measurement error. The CID is a related concept that shows how much change can be deemed as clinically important [23]. That is, CID is the threshold score that a group of patients perceive as noticeable. The MDC and CID facilitate the interpretation of treatment outcomes. For example, the study by Lin et al [24] reported that a true change in the SIS mobility subscale that occurs after rehabilitation needs to show an increase of at least 15.1 points or the change is likely due to an error in the measurement.

In some instances, CID scores do not exceed the MDC scores but still convey information about whether a patient group experienced a clinically important change. In the study of Plummer et al [25], for example, the improvement of 0.11 m/s in gait speed was lower than the measurement error of 0.17 m/s reported by Evans et al [26], indicating that the improvement of gait speed might not be real and beyond measurement error. However, the change of 0.11 m/s gait speed in the Plummer et al [25] study indicated that this patient group improved from the category of physiologic ambulatory to that of full-time home ambulatory, according to the walking categories developed by Perry et al [27]. Without these important benchmarks against which the clinical interpretation is based, clinicians may make erroneous conclusions about the effect of a treatment.

Therefore, this study sought to establish the MDC and CID score estimates of the SS-QOL subscales and assess the proportion of patients' change scores on the SS-QOL subscales that exceeded the MDC and CID in a cohort of patients with stroke who received rehabilitation therapies.

## Methods

### Participants

The study protocol consisted of 2 parts. First, the CIDs data were obtained from participants in randomized controlled trials investigating the effects of 2 upper limb training programs [28,29]. These participants were consecutively screened and recruited from 4 stroke rehabilitation units. Of 126 patients receiving the intervention in these 2 randomized controlled trials, 74 completed the SS-QOL and were included in the present study. The second part of the study is related to MDC. To obtain the test-retest reliability for calculating MCD [30], we recruited 25 patients with chronic stroke from another independent sample.

The inclusion and exclusion criteria for these 2 samples (74 patients for part 1 and 25 samples for part 2) were the same. The inclusion criteria of this study include: first-ever stroke, at least 6 months' poststroke, demonstration of Brunnstrom stage III or higher for the proximal part of the affected upper limb [31], no serious cognitive deficits (score >24 on the Mini Mental-State Exam) [32], and no excessive spasticity at any joint of the upper limb (score of ≤2 on the Modified Ashworth Scale) [33].

Excluded were patients with physician-determined major medical problems and severe aphasia that could potentially confound the study results. This study was approved by Chang Gung Memorial Hospital Human Research Ethics Board (96-0252B) and National Taiwan University Hospital Research Ethics Committee (200903080R), and all participants signed the informed consent forms.

### Interventions and Procedures

Only the 74 participants received 1 of the 3 rehabilitation programs: bilateral arm training (BAT), distributed constraint-induced therapy (CIT), or conventional rehabilitation. Therapy in the BAT group emphasized simultaneous movement of the affected and the unaffected upper limb. The distributed CIT group focused on restriction of movement of the unaffected limb and intensive training of the affected limb. The conventional rehabilitation group focused on neurodevelopment techniques with an emphasis on functional task practice, when possible. The interventions were provided at the participating hospitals under the supervision of 3 certificated occupational therapists. The raters were blinded to the participant group and trained to properly administer the outcome measures. Rater competence was assessed by a senior certified occupational therapist. The same rater administered the SS-QOL evaluation at the 2 different time points (baseline and after the 3-week intervention) for each participant.

### Outcome Measure

The SS-QOL contains 12 subscales (as detailed earlier) with a total of 49 items derived from a series of focused interviews with 34 ischemic stroke survivors [13]. Scoring of the SS-QOL concerns the past week and is rated on a 5-point Likert scale. Response options are scored as 5 ("no help needed/no trouble at all/strongly disagree"), 4 ("a little help/a little trouble/moderately disagree"), 3 ("some help/some trouble/neither agree nor disagree"), 2 ("a lot of help/a lot of trouble/moderately agree"), and 1 ("total help/could not do it at all/strongly agree"). The SS-QOL provides domain scores and a summary score, with higher scores indicating better function. The test-retest reliability, internal consistency, construct, and convergent validity of the SS-QOL have been ascertained in patients with stroke [10,18,21].

Furthermore, the Chinese version of SS-QOL demonstrated adequate Rasch separation reliability and unidimensionality [34]. Because the intervention focused on the rehabilitation of the paretic arm and the improvement of daily functioning, the CID scores based on physical-related subscales directly reflect the benefit of motor intervention. As a result, we only reported the MDC_95 _and CID of the SS-QOL subscales that are related to physical function, including mobility, self-care, and UE function [35].

### Data Analysis

#### Estimation of MDC

The MDC is calculated by multiplying the standard error of measurement (SEM) by 1.96 to correspond to the 95% confidence interval and the square root of 2 to adjust for sampling from 2 different measurements [36]. The SEM is estimated as the pooled standard deviation (SD) of test-retest assessments multiplied by the square root of (1 - *r*), where *r *is the intraclass correlation coefficient (ICC) [37]. The ICC, a kind of test-retest reliability, was determined using a set of independent data from 25 patients in whom the SS-QOL assessment was conducted 2 weeks apart. The ICC was calculated using a 2-way mixed effect model, with a consistency coefficient. MDC_95 _means one can be 95% confident that a change score equal to or exceeding this threshold is true and reliable and not just measurement error [23].

#### Estimation of CID

The distribution-based and the anchor-based approaches were both used to determine the CIDs of the subscales of the SS-QOL. The distribution-based CID estimate was determined using the between-participant baseline SD and the SEM within-participant methods to estimate the CID scores [38]. An effect size is a standardized measure of change over time and represents individual change in terms of the number of pretest SDs. For example, an effect size of 0.5 indicates an increase of 0.5 SD. Cohen [39] has provided benchmarks that serve to guide the interpretations of effects size. According to Ringash et al [40], CIDs are generally close to an effect size of 0.2, and an effect size of 0.5 represents humans' limitation in discrimination [41]. We chose 0.5 SD units to estimate the minimal threshold of CIDs. The SD varies with the heterogeneity of the sample and does not take patient variability of change into consideration. The SEM, which simultaneously incorporates both the sample's reliability and variability into the formula and is relatively sample-independent, is used as another indicator of minimal CID [37].

The anchor-based CID estimate was calculated as the mean change score on each SS-QOL subscale, corresponding to patients who perceived overall increased recovery of 10% to 15% in the Stroke Impact Scale (SIS). We chose SIS as the anchor during the calculation of CID estimates because the overall recovery ratings on SIS directly reflect the participant's viewpoint on the health-related recovery [42,43].

Although there is no defined range of the change score as to the determination of the CID group, several previous studies have found the smallest change score of 10% on the 100-mm visual analog scale (VAS) of quality of sleep [44], 11% on the 100-point Pediatric Evaluation of Disability Inventory (PEDI) [45], and 15% on the 100-mm VAS of back pain [46]. In addition, Duncan et al [47] suggested the clinically meaningful improvement of the SIS global rating scale is within 10% to 15% change. Therefore, patients in the current study were classified into the CID group if a 10% to 15% change was documented on their perceived overall recovery from pretreatment to posttreatment and were considered as having experienced a clinically important change.

Furthermore, to assess the extent of patients' changes after interventions detected by the SS-QOL subscales, the percentage of scale width was calculated by dividing the MDC and CIDs by the total score range of each physical category. For example, the score range of the mobility subscale was from 6 to 30, the total score range of the mobility subscale was 24. In addition, the proportions of patients with change scores greater than the MDC_95 _values and the minimal threshold of CID estimates were calculated.

## Results

Table 1 presents the demographic and clinical characteristics of the 74 patients enrolled in this study as well as the additional 25 patients from the independent sample for calculating test-retest reliability. All characteristics were comparable between these 2 samples, and there were no preexisting differences between the 2 samples on any of the variables.

**Table 1:** Demographic and baseline clinical characteristics of the participants

Characteristic	**n**^a ^**= 74**	**n**^b ^**= 25**	*P*
Age, mean (SD) y	57.1 (11.7)	52.9 (11.2)	0.89^c^
Gender, Male/Female, No.	52/22	17/8	1.00^d^
Months after stroke, mean (SD)	18.1 (16.4)	15.5 (12.8)	0.82^c^
Side of stroke, Right/Left, No.	38/36	16/9	0.25^d^
Stroke subtype, Hemorrhagic/Ischemic, No.	28/46	12/13	0.49^d^
Brunnstrom stage of proximal UE, median (range)	4.5 (3-6)	4 (3-6)	0.61^d^
Fugl-Meyer Assessment UE scores, mean (SD)	44.0 (13.1)	40.8 (14.1)	0.23^c^
Mini Mental-State Exam scores, mean (SD)	27.5 (2.3)	26.6 (2.8)	0.23^c^
Intervention, No.			
Bilateral Arm Training	22		
Constraint-Induced Therapy	16		
Conventional Rehabilitation.	36		

Abbreviations: SD, standard deviation; UE, upper extremity.

^a ^The participants used for estimating clinically important differences; ^b ^The participants used for estimating the test-retest reliability; ^c ^The two-sample *t*-test, 2-tailed; ^d ^Chi-square.

As indicated in Table 2 the MDC_95 _of the mobility, self-care, and UE function subscales were 5.9 (24.6% scale width), 4.0 (20.0% scale width), and 5.3 (26.5% scale width), respectively. According to anchor-based and distribution-based methods, we suggest the respective group-level CIDs for these 3 subscales are in range of 1.5 to 2.4 (6.3% to 10% scale width), 1.2 to 1.9 (6.0% to 9.5% scale width), and 1.2 to 1.8 (6.0% to 9.0% scale width) for the mobility, self-care, and UE function subscales, respectively. As reported in Table 3 an estimated 9.5%, 6.8%, and 12.2% of the patients had a positive change that exceeded the MDC_95 _of the mobility, self-care, and UE function subscales, and 28.4%, 28.4%, and 33.8% of patients' change scores exceeded the lower bound of CID ranges of the mobility, self-care, and UE function subscales, respectively.

**Table 2:** Threshold values of the MDC_95 _and clinically important difference (CID) of the SS-QOL subscales

Subscale	Score range	ICC (95% CI)	MDC_95 _(% scale width)	Distribution-based CID		Anchor-based CID (% scale width)
					
				0.5 SD (% scale width)	1 SEM (% scale width)	
Mobility	6-30	0.84 (0.63, 0.93)	5.9 (24.6%)	2.4 (10%)	1.7 (7.1%)	1.5 (6.3%)
Self-care	5-25	0.88 (0.73, 0.95)	4.0(20.0%)	1.9 (9.5%)	1.2 (6.0%)	1.3 (6.5%)
UE function	5-25	0.88 (0.72, 0.95)	5.3(26.5%)	1.8 (9.0%)	1.3 (6.5%)	1.2 (6.0%)

Abbreviations: CI, confidence interval; ICC, intraclass correlation coefficient; MDC_95_, minimal detectable change at 95% confidence; SD, standard deviation; SEM, standard error of measurement; UE, upper extremity.

**Table 3:** Number of participants who met the criteria of the MDC_95 _and clinically important difference (CID)

Subscale	MDC_95_	Distribution-based CID		Anchor-based CID
		0.5 SD	1 SEM	
	**No. (%)**	**No. (%)**	**No. (%)**	**No. (%)**
Mobility	7 (9.5%)	15 (20.3%)	21 (28.4%)	21 (28.4%)
Self-care	5 (6.8%)	21 (28.4%)	21 (28.4%)	21 (28.4%)
UE function	9 (12.2%)	25 (33.8%)	25 (33.8%)	25 (33.8%)

Abbreviations: MDC_95_, minimal detectable change at 95% confidence; SD, standard deviation; SEM, standard error of measurement; UE, upper extremity.

## Discussion

To the best of our knowledge, this is the first study to determine the MDC and CID scores of the SS-QOL subscales that can be used to differentiate patients treated with stroke rehabilitation who experience real improvement and clinically meaningful change from those who do not. Our findings suggest that a patient's change score has to reach 5.9, 4.0, and 5.3 on the mobility, self-care, and UE function subscales to indicate a true change. That is, when the change scores between the patient's 2 measurements (e.g., baseline and follow-up) reach 24.6%, 20.0%, and 26.5% of the scale width on the mobility, self-care, and UE function subscales, the clinicians may interpret the changes in that patient as true and reliable, given the 95% confidence level.

There is no universally accepted standard for determining the CID [48-52]. An integrated system for defining CID is recommended that combines anchor-based and distribution-based methods [48]. The value and limitations of anchor-based and distribution-based methods in estimating CID have been recognized. The anchor-based approach emphasizes the primacy of a patient's perspective, but anchor-based CID scores may vary with demographic characteristics such as age [49]. Although the distribution-based CID scores are easy to generate, these SD-based scores are associated with some bias due to sample heterogeneity [38]. As a result, a number of recent clinical reports have advocated an approach that combines the anchor-based and distribution-based methods to refine the range of CID [24,50,51].

Using a 1 SEM distribution-based approach, we found that the CIDs for the mobility, self-care, and UE function subscales are 1.7 (7.1% scale width), 1.2 (6.0% scale width), and 1.3 (6.5% scale width), respectively. The SEM incorporates a sample's variability and the reliability of the instrument. Several previous studies have shown that 1 SEM is close to the estimate of CID [53-56]. Despite being theoretically constant [56], the SEM may become larger with a low reliability [57].

Furthermore, the CID scores using 1 SEM would be always less than the MDC values mathematically. Therefore, values of 0.5 SD were calculated as supportive information for determining the CID. On the basis of the 0.5 SD approach, we found that the CID scores for the subscales were 2.4 (10% scale width) for mobility, 1.9 (9.5% scale width) for self-care, and 1.8 (9% scale width) for UE function.

The CID values produced by the anchor-based method were 1.5 (6.3% scale width) for mobility, 1.3 (6.5% scale width) for self-care, and 1.2 (6.0% scale width) for UE function. These estimates were comparable with those obtained from the distribution-based approaches. Because a cutoff threshold of the group-level CID may potentially undermine the clinical interpretation of trial data [58], we reported ranges rather than a single value. We found the CID ranges were 1.5 to 2.4 for mobility, 1.2 to 1.9 for self-care, and 1.2 to 1.8 for UE function. That is, patients with stroke who achieve mean scores in the ranges of 6.3% to 10.0%, 6.0% to 9.5%, and 6.0% to 9.0% of the scale width on the mobility, self-care, and UE function subscales are likely to have clinically meaningful change in these domains.

Of note, there is a concern about the differences between group and individual clinical importance [59]. Average effects across a group may not be meaningful to the individual patient. Group-derived CID values are suitable to interpret the results of clinical trials or group studies, but they are often directly applied to interpret the individual's change [59]. For individual-level use, it may be reasonable to expect that the MDC would be less than or equal to the minimal CID. However, some researchers have suggested that this is not always the case [24,60], which is also consistent with our current findings. When the MDC exceeds the minimal CID, the change score reaching a CID does not mean that patients have exceeded the measurement error, and both values are suggested to be considered in clinical decision making [61].

Taking our cohort sample of stroke rehabilitation as an example, the mean change scores on the mobility, self-care, and UE function subscales were 3.5, 2.8, and 4.1 points, which exceeded the minimal CID ranges. This indicated that the improvements achieved after rehabilitative therapies in this cohort were meaningful to the patients. A mean change score of 1.2 on the self-care subscale in a previous study of the Chronic Disease Self-Management course [17] was reported to achieve statistical significance. This improvement at the group level failed to achieve the lower bound of the minimal CID range established by our current study, which may weaken the validity of the study conclusion about the effect of the self-management education on the quality of self-care after stroke.

Although the validity of a self-rated global assessment scale has been criticized for its "retrospective bias" [50,62,63], we recognized that clinical interpretation of the MDC and CID scores would be enhanced if a patient-driven anchor were included in the study design. Therefore, the reliable-change approach, as proposed by Davidson and Keating [64], was adopted to expand the clinical application of the MDC_95 _and CID established by the current study. The reliable-change approach addresses the question about the proportion of patients exceeding the threshold of MDC and CID. The concept is similar to the event rate, which represents the number of people in whom an event is observed [65]. For example, the event rate is 40% if 40 of 100 patients experience an adverse event such as side effect. On the basis of our results, 9.5%, 6.8%, and 12.2% of patients achieved functional improvement beyond measurement error on the mobility, self-care, and UE function subscales. The greatest proportion of patients that exceeded the lower bound of the minimal CID was observed for the UE function subscale (33.8%), followed by the self-care (28.4%) and mobility (28.4%) subscales. According to Schmitt and Fabio [66], the better the responsiveness of a scale is, the greater the numbers of patients who will exceed the minimal change criteria. Thus, the UE function subscale appears the most responsive subscale among those in the physical category of the SS-QOL for the patients of this study. Because the focus of the rehabilitation used in the current study was on the functional recovery of the paretic arm, it is also possible that the intervention effect was responsible for the relatively greater proportion of patients who exceeded the MDC and CID of the UE function subscale. Further research using larger samples is needed to validate the findings.

It is important to note that the participants included in this study were assigned to receive different treatment programs; thus, the variance in the change scores might have varied among the different treatment groups. Additional analyses of the CIDs for each intervention group showed that the differences in CID values represented by 1 SEM between the participants of each intervention group and the overall participants were less than 0.6 points in the mobility subscale (each intervention participants: 1.3-2.3; vs. overall participants: 1.7) and 0.4 point in the self-care (1.1-1.6 vs. 1.2) and UE function subscales (1.1-1.7 vs. 1.3); and the differences in CID values represented by 0.5 SD between each intervention group participants and the overall participants were less than 0.7 points (mobility: 1.8-3.1 vs. 2.4, self-care: 1.6-2.4 vs. 1.9, and UE function: 1.6-2.5 vs. 1.8). Generally speaking, the CID values in each intervention group are arguably close enough to allow collapse of data from all intervention groups into one group for analysis in each subscale. Given the above information and the fact that the same amount of treatment duration and intensity were used across the different treatment programs, we felt the method of collapsing the data from various intervention groups would be justifiable. For example, some recent studies [67,68] have combined the data from different intervention groups for clinimetric analyses.

The current investigation has some limitations that warrant consideration when interpreting and generalizing the study findings. First, the generalizability of the current findings might be limited. Because we only included patients from departments of rehabilitation with the demonstration of Brunnstrom stage III or higher for the affected UE, the current findings may not be suitable for stroke patient at a Brunnstrom stage of less than III. In addition, some patients were excluded from the current investigation due to cognitive difficulties. To increase the external validity of the results of this study, it is warranted to recruit a wider sample of patients with stroke with differing levels of motor impairment and cognitive difficulty.

Second, because of the relevance of proxy reports for QOL outcome evaluations, particularly in patients with stroke with language impairments [69], there is a need for extended research on the clinimetric properties of the proxy version of the SS-QOL to establish the minimal significant change perceived by the proxies.

Third, although patients who have received different treatment programs with the same treatment duration are often pooled together for clinimetric analysis of the outcome measures [67,68], further research is needed that may investigate the MDC and CID of the SS-QOL for specific interventions based on larger samples to provide further insights into the clinimetric properties of the SS-QOL in specific contexts.

Finally, there are potential clinimetric differences in patient-reported QOL outcomes due to the modes of administration [70]; thus, further research may study clinimetric attributes of the SS-QOL administered in different modes, such as paper-and-pencil administration vs. telephone interviews vs. Web-based electronic data collection.

## Conclusions

In addition to providing information about the psychometric properties of the SS-QOL subscales, the preliminary results of the MDC and CID of the SS-QOL subscales established by this study facilitate the interpretation of the change scores observed in patients with stroke receiving rehabilitation therapies. We found that a patient's change score has to reach 5.9 (24.6% scale width) on the SS-QOL mobility, 4.0 (20.0% scale width) on the self-care, and 5.3 (26.5% scale width) on the UE function subscales to indicate a true and reliable improvement. If the mean change scores for the SS-QOL subscales within a stroke patient group are 1.5 to 2.4 (6.3% to 10% scale width) for mobility, 1.2 to 1.9 (6.0% to 9.5% scale width) for self-care, and 1.2 to 1.8 (6.0% to 9.0% scale width) for UE function, the changes may be considered clinically important. According to the proportions of patients who met the MDC and CID criteria, the UE function subscale seems more responsive than the mobility and self-care subscales for the patients of this study. This may be related to the nature of the rehabilitation therapies involved in our research (i.e., physical interventions that emphasized UE function). Findings of the present study warrant further study based on larger samples involving different types of stroke rehabilitation programs.

## Competing interests

The authors declare that they have no competing interests.

## Authors' contributions

KCL conceived the study, participated in its design and coordination, and helped to draft the manuscript. TF participated in the design of the study, performed the statistical analysis, and participated in the writing of the manuscript. CYW contributed to secure the research funding, designed and conducted the study, and participated in the data interpretation. CJH contributed to the revision of the manuscript. All authors read and approved the final manuscript.
